# Building pragmatic transplant immunobiology in Sri Lanka: a concise review with an LMIC lens

**DOI:** 10.3389/frtra.2026.1772174

**Published:** 2026-04-13

**Authors:** Anura Priyantha Hewageegana

**Affiliations:** Division of Nephrology, National Hospital of Sri Lanka, Colombo, Sri Lanka

**Keywords:** CDC crossmatch, donor-specific antibody, HLA, kidney transplantation, single-antigen bead, Sri Lanka, transplant immunobiology, virtual crossmatch

## Abstract

Sri Lanka's kidney transplantation programme has matured within a resource-constrained public health system and has achieved short-term outcomes that appear comparable to published ranges from similar middle-income programmes, despite major limitations in real-time transplant immunobiology support. However, increasing recipient sensitisation, prolonged dialysis vintage, and a growing deceased-donor programme demand more reliable immunological risk assessment to improve equity and graft outcomes. This pragmatic narrative review summarises the minimum immunobiology package that can support safer kidney transplantation in low- and middle-income settings, using Sri Lanka as an example. Key concepts are explained in simple operational terms, including sensitisation and antibody screening (PRA, including the Zora assay), single-antigen bead testing and donor-specific antibody interpretation, practical use and limitations of virtual crossmatch when donor HLA data are incomplete, and the continuing role of CDC crossmatch where flow-cytometry crossmatch is unavailable. The review also highlights why unexpected early rejection may still occur even when HLA-based testing appears reassuring, and why over-reliance on any single assay can be misleading. Finally, a staged roadmap is proposed—prioritising feasible upgrades, quality assurance, workforce development, and national coordination—to progressively strengthen transplant immunobiology while preserving affordability, fairness, and sustainability. This is a pragmatic narrative review informed by consensus guidance and key peer-reviewed literature, synthesised to prioritise actionable, scalable immunobiology components for low- and middle-income settings.

## Introduction

Kidney transplantation has been established in Sri Lanka for over four decades. Living-donor kidney transplantation developed first, with deceased-donor kidney transplantation introduced approximately two decades later. At present, Kidney transplantation constitutes the predominant solid-organ transplant activity in the country.

The government sector currently operates 13 active kidney transplant centres distributed across the island. In parallel, approximately 10 private hospitals undertake kidney transplantation, almost exclusively within living-donor pathways. Nationally, an estimated 350–400 kidney transplants are performed each year. Importantly, all deceased-donor kidney transplants are performed within government institutions, while private-sector practice is largely confined to living-donor transplantation. Combined organ transplantation and other multi-organ transplant programmes are not routinely performed.

These activity and outcome estimates are derived from aggregated, centre-reported figures rather than registry-verified national data and are presented as contextual benchmarks only.

Outside renal transplantation, liver transplantation is the second most frequently performed solid-organ transplant procedure. Heart transplantation has been undertaken only rarely, and lung transplantation remains exceptionally uncommon, with only one or two procedures performed to date.

To support deceased-donor organ donation and retrieval logistics, Sri Lanka is organised into two principal retrieval zones—the Colombo retrieval zone and the Kandy retrieval zone—which provide the operational framework for donor identification, retrieval coordination, and inter-centre organ transport.

Despite resource constraints, clinical teams have achieved short- and medium-term outcomes that are within ranges reported from comparable middle-income transplant programmes (acknowledging that national metrics are derived from aggregated centre-reported estimates in the absence of a fully operational registry) through careful donor-recipient selection, pragmatic immunosuppression strategies, and close post-transplant monitoring. However, access to advanced transplant immunobiology testing (comprehensive HLA typing, solid-phase antibody assays, and more sensitive crossmatch methods) remains limited within the public programme (1–3). Meanwhile, the epidemiological and service landscape is changing. Sri Lanka's chronic kidney disease (CKD) burden—driven predominantly by non-communicable diseases, with a smaller contribution from CKD of unknown aetiology (CKDu)—is rising. The transplant population increasingly includes candidates with prior transplantation, long dialysis vintage, and sensitising exposures such as multiple pregnancies and blood transfusions, all of which increase alloimmune risk. In parallel, incremental gains in deceased donation and the maturation of a national allocation framework are bringing more complex immunological decisions into routine practice, including re-transplantation and graft offers to highly sensitised recipients (1, 4–8). Against this backdrop, Sri Lanka faces the dual challenge of modernising transplant immunobiology services while preserving equity, affordability, and sustainability in a resource-limited public health system. The country relies on a single central laboratory that provides a restricted menu of tests for the national programme. Donor and recipient HLA typing is typically low-resolution and limited to HLA-A, -B, and -DR, which reduces the reliability of virtual crossmatch (VXM), especially for class II antibodies. Access to single-antigen bead (SAB) testing is sporadic and often associated with long turnaround times; procurement constraints may also result in incomplete antigen coverage or occasional discordant results across platforms. Flow cytometry crossmatch (FCXM) is not available, and a prospective T-and B-cell complement-dependent cytotoxicity crossmatch (CDCXM) is performed before each kidney transplant as the principal physical crossmatch assay (2). This review provides a concise, practice-oriented overview of key transplant immunobiology concepts and translates them into a pragmatic, stepwise model for Sri Lanka and comparable low- and middle-income settings. The aim is not to reproduce textbook histocompatibility detail or propose an idealised system that is financially or logistically unattainable. Instead, we prioritise the investigations that most influence patient outcomes, describe a realistic minimum standard of care, and outline a credible pathway from basic to more advanced immunological capability. Particular emphasis is placed on PRA testing, SAB-based donor-specific antibody (DSA) profiling, VXM, and the role of non-HLA and molecular approaches in selected complex phenotypes (2).

By explicitly framing the discussion through an LMIC lens and drawing on Sri Lankan real-world experience, this review aims to stimulate structured dialogue between clinicians, laboratory teams, health planners, and policymakers. The ultimate goal is to support safe expansion of both living and deceased donor transplantation through a robust, context-appropriate transplant immunobiology service (2).

### Core concepts in transplant immunobiology

We first summarise the foundational pre-transplant tools (PRA/cPRA, SAB, VXM and physical crossmatch), then briefly highlight emerging adjuncts (non-HLA and molecular diagnostics) that explain residual risk.

Molecular diagnostics (biopsy transcriptomics, urine/blood gene-expression profiling, donor-derived cell-free DNA) can improve diagnostic reproducibility and add biological specificity when histology is ambiguous, including some C4d-negative ABMR phenotypes (9–15). Sri Lanka lacks routine access to clinical-grade molecular diagnostics in the public transplant programme. Key barriers include platform and QA costs, sample-handling logistics, bioinformatics capacity, and limited local validation in South Asian populations and high infection-burden settings. A pragmatic pathway is to strengthen the basics first (high-quality biopsy reporting, C4d capability, robust HLA typing, reliable DSA workflows) and introduce molecular tools through staged pilots or research collaborations aligned to clear clinical questions and outcome audit (9–15).

### Transcriptomic and molecular diagnostics: future-facing, but not first-line priorities

Non-HLA antibodies (e.g., MICA, AT1R and other endothelial/podocyte targets) can contribute to graft injury even when HLA DSA are not detected, and NK-cell pathways (including “missing self”) may modulate injury phenotypes (16). In an LMIC setting, these concepts are most useful to explain disproportionate early dysfunction when standard HLA-focused testing appears reassuring, particularly where donor typing is incomplete. The practical message is to avoid HLA-centric overconfidence: a negative CDCXM and/or a “clean” SAB panel does not exclude accelerated immune injury. Where feasible, biopsy and careful clinicopathological correlation remain central (16).

### Non-HLA antibodies and “missing self”: why “negative” tests do not equal “no risk”

In Sri Lanka, SAB-based DSA profiling is not consistently available and may be intermittent, leading to constraints such as outdated antibody profiles, difficulties comparing across platforms/lots, and absence of complement-binding (C1q/C3d) or IgG subclass testing (17). A pragmatic approach is to prioritise: (i) identifying clearly positive, reproducible DSA (especially class II), (ii) avoiding high-risk DSA when alternatives exist (particularly in first transplants), and (iii) in unavoidable-risk situations (re-transplant/highly sensitised candidates), planning peri-operative strategy and early post-transplant monitoring with a low threshold for biopsy when graft function is suboptimal.

Pre-formed DSA—especially persistent class II antibodies and complement-binding DSA—are associated with higher rates of ABMR and inferior graft survival; *de novo* DSA post-transplant similarly predict chronic ABMR and allograft loss (1, 2, 4, 5, 17–19). Practical interpretation should consider: antibody class (I vs. II), locus (e.g., DQ), strength and trends over time, assay artefact risk, and the clinical setting (17–20). A single high MFI value is insufficient on its own; longitudinal patterns and clinicopathological correlation are more informative (18, 20).

### Donor-specific antibodies (DSA): interpreting what matters

In high-income settings, VXM can replace routine prospective crossmatch in many scenarios, improving logistics without compromising safety when donor typing is comprehensive and antibody data are current (14, 21). Flow cytometry crossmatch (FCXM) is more sensitive than CDCXM and can refine risk in complex cases, but it is technically demanding and often unavailable in LMIC programmes (21). In Sri Lanka, prospective T- and B-cell complement-dependent cytotoxicity crossmatch (CDCXM) remains the principal physical crossmatch before transplantation. While less sensitive than FCXM and likely to miss low-level DSA, CDCXM can detect strong complement-fixing antibodies associated with hyperacute or early severe ABMR, and it provides a pragmatic safeguard when VXM is limited by incomplete donor HLA data or constrained SAB access (2, 21).

### Virtual crossmatch (VXM), CDCXM, and where FCXM fits

We acknowledge an important limitation relevant to many low- and middle-income country (LMIC) settings, namely the under-representation of local and South Asian HLA alleles in currently available virtual crossmatch platforms and commercial bead panels (14, 21–25). This may complicate antibody assignment and crossmatch interpretation, particularly when donor typing is limited to low-resolution HLA data or when class II loci are incompletely characterised (14, 21). In such contexts, including countries such as Sri Lanka and India, a physical crossmatch remains an essential safety step despite the use of algorithm-driven virtual crossmatching (14, 21). We note that this limitation may be mitigated in the future as expanded-coverage kits, locally validated allele datasets, and epitope-based analytical approaches become more routinely available; however, at present it represents a practical constraint that influences the design and implementation of national allocation algorithms (14).

### LMIC allele under-representation and the continuing need for physical crossmatch

Single-antigen bead (SAB) assays enable antigen-specific detection of anti-HLA antibodies and provide a semi-quantitative signal (mean fluorescence intensity, MFI). SAB underpins DSA profiling, VXM, and cPRA calculation (2, 3, 18–20, 22, 26, 27). However, SAB is vulnerable to clinically important interpretive pitfalls (e.g., prozone/complement interference, denatured antigen reactivity, lot-to-lot variation), and “MFI” is not a direct measure of pathogenicity (18–20, 28, 29). In resource-limited systems where repeat testing, dilutions, EDTA treatment, or parallel platforms may be unavailable, structured reporting and clinician–laboratory communication become essential to avoid over-interpretation (18–20, 28, 29).

### Solid-phase assays (SAB) and practical pitfalls

In Sri Lanka, CDC-based PRA using limited donor lymphocyte sets (Zora assay) remains routine, while systematic cPRA is not yet integrated into national workflows. Accurate cPRA requires: (i) reliable antibody specificity assignment (typically via SAB), (ii) appropriate donor-pool HLA frequency data, and (iii) agreement on thresholds for designating antigens “unacceptable” for allocation (6–8). Even partial implementation—focused on re-transplant and highly sensitised candidates—could improve allocation efficiency and reduce futile offers (6–8).

PRA historically estimated the breadth of sensitisation as the percentage of a donor panel expected to be incompatible. Classical cell-based PRA (e.g., CDC assays against lymphocyte panels) is labour-intensive, panel-dependent, and poorly standardised (6–8). With solid-phase assays, sensitisation assessment has evolved toward cPRA, where a candidate's “unacceptable antigens” (based on detected clinically relevant antibodies) are mapped onto donor HLA frequency data to estimate the proportion of donors likely to be incompatible (3, 6–8).

### Panel reactive antibody (PRA) and calculated PRA (cPRA)

Transplant immunobiology aims to identify and mitigate alloimmune risk before and after kidney transplantation, using a limited set of tests that must be interpreted in clinical context. In LMIC settings, the priority is not maximal test coverage but a minimum package that prevents catastrophic incompatibility, supports rational allocation (especially for DDKT), and can be delivered reliably and equitably.

The staged framework is derived from synthesis of published histocompatibility consensus recommendations and the operational constraints described for Sri Lanka, with each stage limited to additions that are quality-assurable and clinically actionable in routine care.

**Figure 1:**
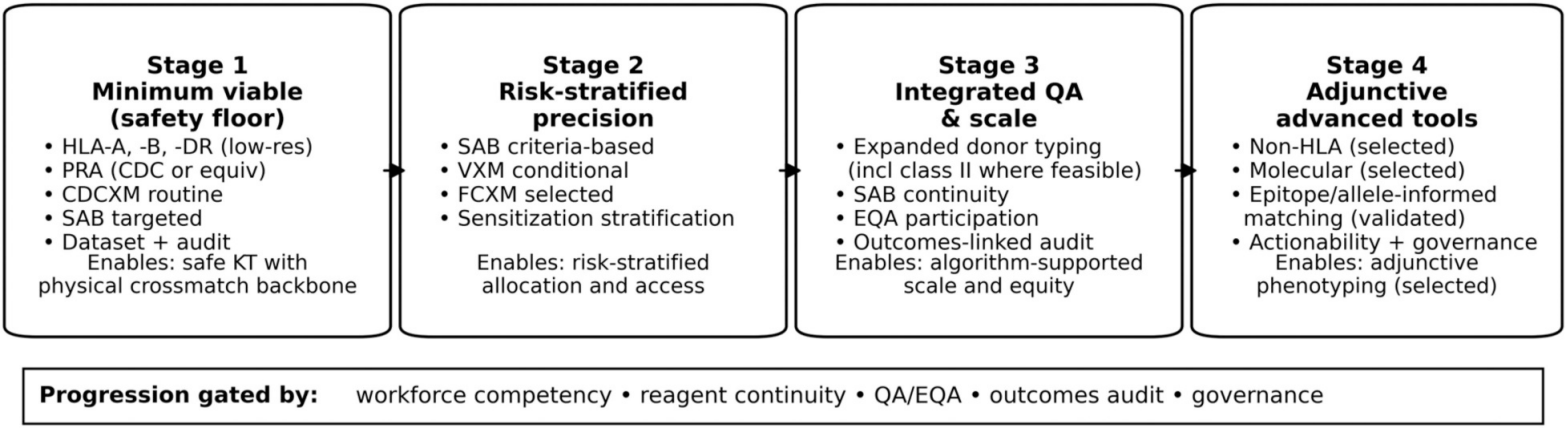
Provides a visual summary of this staged escalation and its dependencies. Staged escalation of transplant immunobiology services for low- and middle-income settings. Stage 1 defines the minimum viable immunobiology package for safe kidney transplantation. Stages 2-4 add precision and programmatic integration as workforce competency, reagent continuity, quality systems (including EQA), audit, and governance mature; advanced assays are introduced only when clinically actionable. CDC, complement-dependent cytotoxicity; CDCXM, complement-dependent cytotoxicity crossmatch; EQA, external quality assurance; FCXM, flow-cytometric crossmatch; HLA, human leukocyte antigen; PRA, panel reactive antibody; SAB, single-antigen bead; VXM, virtual crossmatch.

These principles are operationalised in a staged framework for LMIC transplant programmes (Table 1 and Figure 1).

**Table 1:** Minimum viable transplant immunobiology service for safe kidney transplantation in low- and middle-income settings.

Component	Stage 1 minimum	Primary decision supported	High-impact/mandatory use	Report/QA minimum
Recipient HLA typing	HLA-A, -B, -DR (antigen-level)	Baseline risk classification; listing readiness	All candidates (living and deceased)	Loci/resolution stated; method; internal QC
Donor HLA typing	HLA-A, -B, -DR (antigen-level)	Offer suitability; crossmatch planning	All deceased donors pre-allocation/implantation where feasible	Missing loci declared; turnaround target; delay audit
Sensitization estimate (PRA/cPRA)	PRA (CDC or equivalent) with pragmatic sensitisation category	Risk stratification; testing intensity	All waitlisted; after sensitising events	Method/date; category; validation plan
Antibody specificity (SAB)	Targeted (criteria-based access)	Define unacceptable antigens; compatibility risk	Re-transplant; pregnancy/transfusion; elevated PRA; prior rejection	Platform/date; key specificities; caveats; lot tracking if feasible
Virtual crossmatch (VXM)	Conditional (programme criteria)	Rapid offer triage when prerequisites met	Only when donor typing and recent antibody data are adequate	Assumptions stated; eligibility criteria; governance sign-off
Physical crossmatch (CDCXM)	Routinely available and performed	Proceed/decline when VXM uncertain or donor typing incomplete	Default for deceased donors when VXM prerequisites not met; sensitised/re-transplant	Report T- and B-cell separately; controls; SOPs; competency logs
Physical crossmatch (FCXM)	Stage 2 + expansion (optional)	Refine risk in sensitised/re-transplant or discordant cases	Sensitised/re-transplant; discordant/uncertain results	Positivity thresholds; standard gating; QA log
Non-HLA antibodies	Adjunctive only (selected indications)	Phenotype clarification in selected AMR-like scenarios	Unexplained dysfunction/AMR-like phenotype without HLA-DSA (selected)	Label as adjunctive; governance over indications
Molecular diagnostics	Adjunctive only (selected settings)	Support pathways when results are actionable	Research/selected complex phenotypes	Interpret as adjunctive; actionability and cost justification
Data capture & audit loop	Minimum dataset maintained	Quality improvement; equity monitoring	All transplant episodes	Offer/acceptance, immunology, outcomes; annual feedback loop
Quality assurance/EQA	Phased implementation	Assay reliability and scalability	Core assays as feasible	EQA status declared; roadmap to EQA; incident review

Stage 1 defines the minimum viable immunobiology service for safe kidney transplantation at scale; higher stages represent incremental expansion contingent on workforce capacity, reagent continuity, and quality systems. VXM should not be used as a standalone decision tool when donor typing is incomplete (particularly class II) or antibody data are outdated/limited. CDC, complement-dependent cytotoxicity; CDCXM, complement-dependent cytotoxicity crossmatch; cPRA, calculated panel reactive antibody; EQA, external quality assurance; FCXM, flow-cytometric crossmatch; HLA, human leukocyte antigen; PRA, panel reactive antibody; SAB, single-antigen bead; VXM, virtual crossmatch.

To translate these core principles into implementable practice, we propose a staged framework for transplant immunobiology service development that prioritises safety and scalability (Table 1 and Figure 1).

## The Sri Lankan reality: current capacity and gaps

### Existing infrastructure and service configuration

All transplant-related immunological testing in Sri Lanka is currently centralised within a single national immunobiology laboratory, operating in association with the National Blood Transfusion Service (NBTS). This facility provides transplant immunology support for all government-sector kidney transplant centres nationwide.

Although transfusion medicine specialists are distributed across approximately 25 regional clusters throughout the country, the number of dedicated, trained technical personnel available for transplant immunobiology remains limited. Consequently, the system is not yet resourced to deliver a consistently uninterrupted 24-hour service, a constraint that is most evident in time-sensitive settings such as deceased-donor organ allocation and urgent crossmatch-dependent pathways.

Operational bottlenecks include workforce limitations, intermittent reagent supply, equipment and maintenance constraints, and restricted access to validation and external quality assurance resources. Collectively, these factors constrain the breadth, turnaround time, and scalability of immunological testing and underscore the need for strategic prioritisation of high-impact assays, alongside phased expansion aligned with national transplant activity and service demands.

In the absence of a fully operational national transplant registry, programme-level indicators are currently derived from aggregated, centre-reported activity and follow-up data. Across Sri Lankan centres, overall kidney transplantation is estimated at approximately 350–400 procedures per year, with living donation contributing roughly three-quarters and deceased donation approximately one-quarter of activity. Deceased-donor kidney transplantation has expanded over the past decade, from fewer than 20 procedures annually in the early 2010s to approximately 100 per year in the early 2020s, alongside broader multi-centre participation. Within current activity, re-transplantation accounts for around 5% of procedures and highly sensitised recipients are estimated to comprise approximately 10%–15% of candidates. Aggregated centre-reported outcomes suggest approximately 92% one-year graft survival and approximately 88% five-year graft survival; these estimates should be interpreted cautiously and primarily as contextual benchmarks until systematic national reporting is established.

This national reference laboratory performs baseline HLA typing for donors and recipients, limited SAB testing, and prospective T-and B-cell CDC crossmatch (CDCXM) before each kidney transplant. Current HLA typing is low-resolution (antigen-level) and largely restricted to six antigens at HLA-A, -B, and -DR; class II typing is effectively limited to DR (without routine DQ or DP testing). From the nephrologists’ perspective, upgrading to at least 4-digit (allele-level) typing -especially including class II DQ (DQB1, and ideally DQA1) -is earnestly required to improve antibody interpretation, strengthen the reliability of virtual crossmatch decisions, and support better transplant outcomes as the programme expands. Turnaround times for more complex assays may be prolonged, reflecting both logistical and staffing constraints. Key characteristics of the current configuration include:

- No FCXM capability;
- No systematic cPRA calculation integrated into allocation workflows;
- SAB testing available on an *ad hoc* or case-by-case basis, with variable panel completeness; and
- Limited access to non-HLA antibody testing, and no access to molecular diagnostics at present. Transplant centres, particularly those outside the capital, may experience delays in obtaining test results, and decisions often rely on a combination of historical clinical risk factors, empirical judgement, and whatever immunological data are available at the time.

### Ground realities and operational constraints

Several ground realities shape what is realistically achievable in the short to medium term:

- Budgetary constraints in the public health system limit the ability to fund high-cost assays and reagents, especially when competing against numerous other priorities in non-communicable and communicable disease control.
- Procurement processes may be slow and inflexible, leading to interruptions in reagent supply and challenges in maintaining consistent kit platforms.
- Human resource limitations constrain the capacity for intensive, labour-heavy assays such as FCXM, as well as for broader quality assurance and external proficiency testing. There is also a risk of adopting advanced tests in a few places without fully integrating them into routine clinical workflows. This can create small “islands of excellence” in the laboratory, yet deliver little population-level benefit because referral pathways, sample transport, reporting, clinical decision-making, and access to treatment are not aligned. As Sri Lanka decides which immunobiology technologies to prioritise, the focus should be on tests that can be implemented reliably at scale and linked to clear clinical actions.

These implementation realities are shared across many LMIC programmes, making the Sri Lankan experience a useful template for phased, scalable immunobiology development.

Having outlined the current national configuration and constraints, the practical question becomes which immunobiology elements should be prioritised first within Sri Lanka's resource envelope.

## Strategic priorities: what truly matters first?

### Implications for LMIC-focused immunobiology development

The Sri Lankan experience highlights challenges common to many LMIC transplant programmes, where transplant volumes are increasing in the context of constrained laboratory infrastructure and workforce capacity. In such settings, transplant immunobiology services must be developed through phased, pragmatic approaches, prioritising essential assays, centralisation of services, workforce development, and alignment of laboratory expansion with realistic transplant activity and governance frameworks.

### Defining a minimum viable immunobiology service

In an LMIC setting, it is neither feasible nor necessary to replicate every aspect of high-income histocompatibility practice. Instead, the goal should be to define a minimum viable immunobiology service that: (1) Prevents catastrophic immunological events (e.g., hyperacute rejection, early severe ABMR); (2) Enables rational allocation and risk stratification, especially in deceased donor programmes; and (3) Can be delivered reliably across the country (not only in a few centres), maintained long term with predictable funding, staff, and supplies, and provides fair access to testing and transplant decision-making regardless of geography or ability to pay. For Sri Lanka, such a minimum service would ideally include:

- Reliable, countrywide HLA typing for at least HLA-A, -B, -DR, and DQ for donors and transplant candidates (recognising that current national typing is antigen-level and typically limited to HLA-A, -B, and -DR);
- Access to SAB-based DSA profiling for all re-transplantation candidates and selected high-risk first transplants;
- Routine incorporation of SAB results into a basic VXM framework to guide acceptance or refusal of donor offers;
- CDCXM capacity for high-risk or equivocal scenarios, with clearly defined indications; and
- Basic infrastructure for data capture and audit, enabling longitudinal tracking of sensitisation, rejection episodes, and graft outcomes. Elements such as FCXM, complement-binding DSA assays, epitope-based matching, and molecular diagnostics should be considered secondary priorities that can be added once this foundation is solid.

### Appropriateness rather than maximalism

A recurring theme in LMIC transplant immunology is the risk of “unnecessary diagnostic escalation”, where sophisticated tests are adopted piecemeal because they are technologically attractive or academically interesting, rather than because they address the most pressing clinical questions. This can lead to inappropriate or inefficient resource use, particularly when basic services remain underdeveloped. Sri Lanka should consciously adopt an appropriateness framework, asking for each proposed technology:

- Does this test answer an important clinical question that cannot be reliably answered with existing tools?
- Will the result of the test change management in a way that is feasible and affordable in our context?
- Can we maintain the quality and consistency of the assay over time, including reagents, equipment, and staff expertise? For example, cPRA calculation may offer substantial value in prioritising highly sensitised candidates in a growing DDKT programme, but only if HLA frequency data and DSA thresholds are locally validated. Similarly, FCXM might improve sensitivity in high-risk crossmatch scenarios, but its incremental benefit over well-performed CDCXM and SAB-based VXM may be modest until the volume of such high-risk cases justifies the investment.

### A pragmatic, staged model for Sri Lanka

Stage 1: Strengthen core HLA typing and structured access to antibody testing (SAB).

- Standardise donor and recipient HLA typing workflows; expand to include DQ when feasible.
- Secure reagent and equipment maintenance funding with defined turnaround targets.
- Define a national minimum immunology dataset and SOPs for listing readiness and offer evaluation.
- Make SAB mandatory for re-transplant candidates and selected high-risk first transplants; repeat after sensitising events.
- Use standardised reporting (platform, date, key specificities, MFI bands, caveats) and lot tracking when feasible.

Stage 2: Implement cPRA (starting with high-risk cohorts) and introduce governance-led virtual crossmatch (VXM).

- Define unacceptable antigens and VXM prerequisites (current antibody profile and adequate donor typing, including class II where possible).
- Audit declined and futile offers, early rejection, and outcomes; refine local thresholds iteratively.

Stage 3: Add FCXM and complement-binding assays selectively, only when results are actionable and quality assured.

- Introduce FCXM for discordant or very high-risk cases once staff competency, QA, and case volume justify it.
- Consider C1q/C3d (and, where available, IgG subclass) testing only if it changes peri-operative strategy or monitoring.

Stage 4: Pilot molecular and non-HLA approaches through collaborations (research or tightly selected phenotypes).

- Run limited pilots of biopsy transcriptomics or donor-derived cell-free DNA for ambiguous/high-risk phenotypes, linked to outcome audit.
- Reserve non-HLA antibody testing for selected unexplained AMR-like phenotypes when results are interpretable and actionable.

## Collaboration, governance, and quality assurance

### National governance and policy integration

To avoid fragmentation and duplication, transplant immunobiology services should be embedded within a clear national governance framework. Essential elements include:

- Designation of the central immunobiology laboratory as a national reference centre with defined responsibilities for quality assurance, training, and policy implementation;
- Integration of HLA and DSA data into transplant programme databases and, where feasible, a future national registry for outcome tracking and programme evaluation (Sri Lanka currently lacks a comprehensive national renal/transplant registry); and
- Inclusion of transplant immunobiology within national kidney health policies (dialysis, CKD prevention, and deceased donation), with explicit standards for transfusion stewardship, referral and listing pathways, minimum donor/recipient immunology datasets and turnaround targets, and mandatory reporting to a national registry for audit and programme improvement (30). Periodic dialogue between nephrology, immunology, pathology, and health ministry stakeholders is crucial to align priorities, allocate resources, and monitor progress.

Practically, registry-enabled data capture would allow systematic recording of sensitisation profiles (including cPRA where implemented), crossmatch and virtual crossmatch outcomes, early rejection phenotypes, infection events, and graft and patient survival. These data are essential for rational planning of immunobiology capacity, prioritisation of high-impact assays, benchmarking of programme performance across centres, and evaluation of each step in staged laboratory expansion. Registry-derived epidemiological insight would also support evidence-based refinement of allocation policy, transfusion stewardship, and workforce planning.

### Collaboration with regional centres

Given the shared challenges faced by many South Asian and LMIC settings, Sri Lanka can benefit from structured collaboration with regional high-capacity centres that have more advanced histocompatibility services. Examples include:

- External quality assurance and proficiency testing for HLA typing, SAB, and crossmatch assays;
- Joint training programmes for laboratory staff and clinicians;
- Access to specialised testing (e.g., complex FCXM, non-HLA assays, molecular diagnostics) for selected cases that cannot be adequately evaluated locally; and
- Participation in regional research networks focused on transplant immunobiology in LMICs. Collaboration must, however, be pragmatic and sustainable. Over-reliance on external laboratories for routine services may undermine the development of local capacity. Instead, Sri Lanka should aim for a balanced model where essential day-to-day testing is performed domestically, while highly specialised assays are accessed via formalised regional referral arrangements.

### Periodic case-based discussions and joint review

Instead of attempting to create formal joint “immunology–nephrology conferences” that may be logistically challenging in the current setup, Sri Lanka can adopt a more pragmatic approach by arranging periodic case-based discussions focused on diagnostically or therapeutically challenging cases. These could involve:

- Detailed review of immunobiology results, histology, and clinical course;
- Shared learning around interpretive pitfalls (e.g., prozone, denatured antigen reactivity, non-HLA phenomena); and
- Development of consensus-based local protocols that evolve with experience. Virtual platforms, teleconferencing, and secure data-sharing channels can facilitate such interactions without requiring substantial additional infrastructure.

### Regional experience to inform local choices

Reports from Indian and other regional centres show that SAB-based antibody profiling, targeted virtual crossmatch (VXM), and algorithmic compatibility workflows can be implemented at scale under resource constraints, provided reporting is standardised and laboratory-clinician communication is strong; common interpretive pitfalls (e.g., prozone, denatured antigen reactivity) need explicit mitigation. While Sri Lanka's smaller volume limits direct extrapolation, these experiences provide a practical template for phased adoption linked to audit of rejection and graft outcomes (23–25).

## A priority-first roadmap for low-and middle-income countries

Although this review is rooted in the Sri Lankan experience, many of the principles and recommendations are relevant to other LMICs facing similar constraints. A priority-first roadmap might be summarised as follows: (1) Get the basics right: universal access to robust HLA typing and at least one reliable crossmatch method (usually CDCXM). (2) Target SAB and DSA testing to those who most need it, using clear national algorithms. (3) Introduce cPRA and VXM gradually, starting with high-risk candidates and high-volume centres, and audit outcomes closely. (4) Consider FCXM, complement-binding DSA, and epitope-based strategies only once core services are stable and widely available. (5) Explore molecular diagnostics cautiously and primarily within research collaborations. (6) Embed immunobiology services within national transplant governance and data systems. (7) Leverage regional collaboration for quality assurance and access to specialised assays, without neglecting local capacity building. Throughout, the focus should remain on appropriateness, sustainability, and equity, resisting the temptation to pursue technological maximalism that benefits a few while diverting resources from essential services.

In comparative terms, Sri Lanka most closely resembles mid-spectrum LMIC programmes in which transplantation is delivered largely within the public sector, but histocompatibility capacity is concentrated in one or a small number of reference laboratories, and expansion is limited by workforce and reagent continuity. Similar patterns have been described in parts of South and Southeast Asia and in selected Latin American settings, where core HLA services and targeted antibody testing are prioritised before broader molecular platforms. By contrast, several programmes in sub-Saharan Africa remain earlier in the development spectrum, with transplantation concentrated in few centres and minimal local immunobiology infrastructure, making regional referral and stepwise adoption of basic assays the dominant strategy. Accordingly, the staged framework proposed here is intended to be adaptable across varying LMIC maturity levels rather than specific to Sri Lanka alone.

## Conclusion

Sri Lanka's kidney transplantation programme has reached a point where incremental, opportunistic enhancements in transplant immunobiology are no longer sufficient. Growing caseloads, increasingly complex immunological profiles, and the expansion of deceased donation demand a more deliberate, structured approach. This review has outlined a pragmatic, staged model that prioritises core HLA typing, SAB-based DSA profiling, cPRA, VXM, and basic crossmatch capability as foundational elements, while positioning more advanced tools—such as FCXM, complement-binding assays, non-HLA antibody testing, and molecular diagnostics— as second-tier enhancements to be adopted judiciously. By defining a minimum viable immunobiology service that is both evidence-based and context-appropriate, Sri Lanka can strengthen the safety and effectiveness of its transplant programme without overextending limited resources. Central to this effort will be: sustained investment in laboratory infrastructure and human resources; robust national governance and data systems; and purposeful collaboration with regional centres and international partners. If these elements can be aligned, transplant immunobiology in Sri Lanka can evolve from a patchwork of tests and practices into a coherent, patient-centred system that supports equitable access to high-quality kidney transplantation—and, in so doing, offers a priority-first roadmap for low- and middle-income countries (LMICs), rather than a textbook aspiration that remains out of reach.

This review is aligned with contemporary consensus and classification updates in kidney transplant pathology and antibody monitoring, including recent Banff meeting reports and current clinical recommendations for post-transplant donor-specific antibody assessment and interpretation (31–33).
